# Pediatric chylolymphatic mesenteric cyst - a separate entity from cystic lymphangioma: a case series

**DOI:** 10.1186/1752-1947-3-111

**Published:** 2009-11-09

**Authors:** Kamal Nayan Rattan, Vimoj J Nair, Manish Pathak, Sanjay Kumar

**Affiliations:** 1 Department of Pediatric Surgery, Pt. B.D.Sharma, Post Graduate Institute of Medical Sciences, Rohtak, Haryana, 124001, India; 2 Department of Radiation Oncology, Pt. B.D.Sharma, Post Graduate Institute of Medical Sciences, Rohtak, Haryana, 124001, India; 3 Department of Pathology, Pt. B.D.Sharma, Post Graduate Institute of Medical Sciences, Rohtak, Haryana, 124001, India

## Abstract

**Introduction:**

Chylolymphatic mesenteric cysts are rare entities with variable presentations and this has surgical implications in the pediatric age group.

**Case presentation:**

We carried out a retrospective analysis of the clinical and histopathological records of pediatric patients diagnosed and treated for chylolymphatic mesenteric cysts at our institute from 1998 to 2008. Eight patients met the histopathological criteria of chylolymphatic mesenteric cyst. These patients were in the age range 18 months to 10 years with a mean age of 4.5 years. Of these eight patients, four presented with an abdominal lump, and two each with abdominal pain and acute intestinal obstruction. On clinical examination, five out of the eight patients had a palpable abdominal mass. Laparotomy and complete excision of the cyst along with the involved gut was performed in all patients. There were no postoperative complications or any recurrence during the follow-up period which ranged from 4 months to 8 years.

**Conclusion:**

Although very rare, chylolymphatic mesenteric cyst should be kept in mind as one of the differential diagnoses of cystic masses of the abdomen including cystic lymphangioma. Ultrasonography and computed tomography suggest the diagnosis but histopathological examination is required for confirmation. Complete excision of the cyst yields excellent results.

## Introduction

A chylolymphatic cyst is a rare variant of a mesenteric cyst [1,2]. These cysts present within the mesentery, lined with a thin endothelium or mesothelium and filled with chylous and lymphatic fluid [3]. Although mesenteric cysts in general have been reported in the literature fairly frequently, chylolymphatic cysts in the pediatric age group are extremely rare in the modern medical literature [2], therefore very little information is available regarding their presentation and complications. We present our unique experience of eight cases during the past 10 years in the hope that this information will reinforce the diagnostic and treatment strategy. This is one of the largest reported case series of chylolymphatic cysts in the pediatric age group.

## Case presentation

Clinical case records at our institute from the period 1998 to 2008 were reviewed for pediatric patients with a diagnosis of chylolymphatic cyst of the mesentery. For the study, an age of 16 years or younger was considered to be in the pediatric age group. The following descriptive data were analyzed: age at presentation, gender, presenting symptoms, duration of symptoms, physical findings, imaging results, pre-operative, peri-operative and postoperative management, operative findings, and complications. The histopathological characteristics of each surgical specimen were also examined. The data obtained were analyzed and compared with the available literature.

## Results

**Figure 1:**
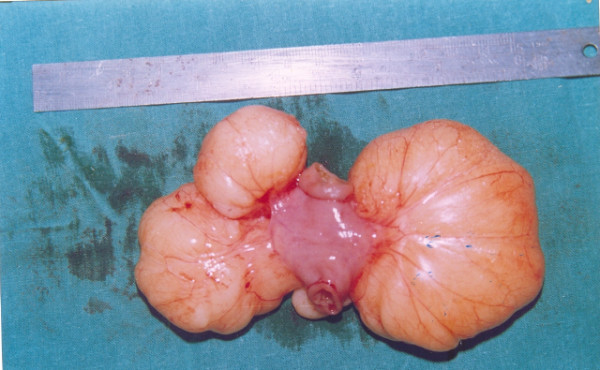
**Pediatric chylolymphatic mesenteric cyst**. Resected specimen of pediatric chylolymphatic mesenteric cyst showing multiloculated cyst along with resected gut.

**Figure 2:**
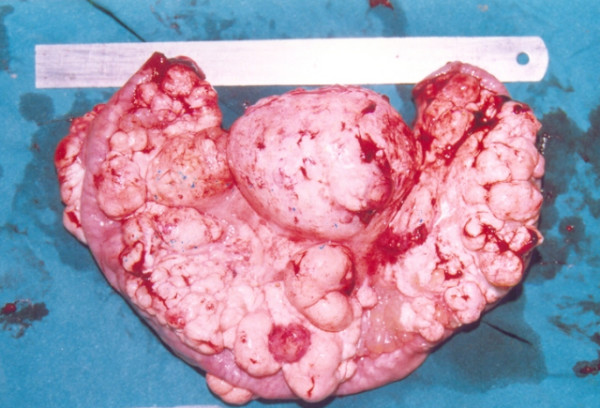
**Pediatric chylolymphatic mesenteric cyst, histopathologic image**. Microphotograph showing cyst wall with endothelial lining and chylous fluid (hematoxylin and eosin stain ×100).

Retrospective analysis revealed eight patients meeting the histopathological criteria of chylolymphatic cyst. The age of the eight patients ranged from 18 months to 10 years with a mean age of 4.5 years. Five patients were male and three were female. Of these eight patients, four presented with an abdominal lump. Of the remaining four patients, two patients presented with an abdominal pain and two with acute intestinal obstruction presenting early, within 2 days of becoming symptomatic. Patients with an abdominal lump and painful abdomen had delayed presentation. Intermittent, colicky pain was poorly localized and was mild to moderate in intensity. Three of the four patients presenting with an abdominal lump and pain also had intermittent episodes of abdominal distension, bilious vomiting and constipation suggesting subacute intestinal obstruction, and required hospital admission elsewhere. On clinical examination, five out of the eight patients had a palpable abdominal mass. In both the patients presenting with acute intestinal obstruction, plain abdominal radiography showed multiple air-fluid levels. A mass defect displacing the bowel loops around it was visible on plain abdominal radiographs in all of the patients. On sonography, all patients showed multiloculated cystic lesions in relation to the gut. Abdominal computed tomography was done in only two cases and revealed similar findings. All our patients underwent elective laparotomy after initial resuscitation. All had a multiloculated cyst involving the small bowel mesentery (Figure 1). The cysts were of varying sizes with the smallest approximately 8 mm in diameter and the largest approximately 9 cm in diameter. In two patients, acute stretching of the bowel loop over the large cyst led to acute intestinal obstruction. Resection of the involved gut along with the cyst was required in all patients as the involved gut shared the vascular supply with the cyst. Postoperative recovery in all patients was excellent. No recurrence was noted during the follow-up period, which ranged from 4 months to 8 years. All specimens were sent for histopathological examination, which revealed multiloculated cysts lined with endothelium and filled with chylous fluid and lymph (Figure 2). Thus, all cases were confirmed to be chylolymphatic cysts by histopathology.

## Discussion

Chylous cysts are rare variants of mesenteric lesions and constitute 7.3% to 9.5% of all abdominal cysts [1]. There are very few cases of pediatric chylolymphatic cysts reported in the literature. Engel *et al*. [1] reported four cases of cystic lymphangioma in the pediatric age group and only two of them were of the chylous variety. Singh *et al*. [4] reported five cases of cystic lymphangioma in the pediatric age group though only one of them was diagnosed to be a chylolymphatic cyst on histopathology. Gupta *et al*. [2] reported a neonate who presented with intestinal obstruction due to a chylous mesenteric cyst. Panjwani *et al*. [5] reported an isolated case of a chylolymphatic mesenteric cyst in a 10-day-old neonate. Ratan and coworkers [6,7] reported a mesenteric cyst causing caecal volvulus. Our series also contained a mesenteric cyst resulting in caecal volvulus. Here we have reported a series of eight cases of chylolymphatic cysts in the pediatric age group with variable presentations: abdominal lumps, acute or subacute intestinal obstruction, and even poorly localized abdominal pain, which resulted in the patient receiving treatment from a private practitioner.

Beahrs *et al*. [8] classified mesenteric cysts into four groups based on etiology: embryonic or developmental; traumatic or acquired; neoplastic; and infective or degenerative. Recently, a pathologic classification system has been proposed [9]. Types 1 (pedicled) and 2 (sessile) are limited to the mesentery and can be excised completely with or without resection of the involved gut. Types 3 and 4 (multicentric) extend into the retroperitoneum and require complex operations and often sclerotherapy as well. Based on the contents of the cyst, the mesenteric cyst can be divided into serous, chylous, hemorrhagic and chylolymphatic cyst. The chylolymphatic cyst, as indicated by its name, contains both chyle and lymph. The accumulation of chyle and lymph is considered to be the result of an imbalance between the inflow and outflow of fluid [1]. This cyst may be asymptomatic, and may cause abdominal distension or an abdominal lump or may present with complications such as intestinal obstruction, hemorrhage, infection, rupture of the cyst, volvulus or obstruction of the urinary or biliary tract. In our study, four of the eight patients presented with an abdominal lump while the lump was clinically palpable in five patients. Painful abdomen and acute intestinal obstruction was the presentation in two patients each.

Radiological investigations form an integral part of the management of these lesions. A plain abdominal radiograph may show a gasless, homogenous mass defect displacing the bowel loops around it. In a child with an obstructed intestine, multiple air-fluid levels will be seen on an erect abdominal radiograph. Barium studies are now only of historical interest; abdominal ultrasonography is currently the imaging procedure of choice. This delineates the nature of the mass, organ or site of the origin, and the extent and associated mass effects on the kidney or liver, if any. In a chylolymphatic cyst, a 'fluid-fluid level' can be seen on ultrasonography due to formation of an upper fluid level by lighter chyle over a lower fluid level of heavier lymph [10]. Computed tomography adds little additional information; however, contrast-enhanced film can show the relationship of the bowel and other vital structures to the lesion. Some authors have described the characteristic appearance of a chylolymphatic cyst on computed tomography in the form of the presence of fluid levels of differing echodensities, that is to say, an upper fatty echodensity of chyle on top of the water echodensity of lymph in a well-defined cystic lesion [10,11].

Antenatal detection of cystic abdominal lesions is possible in a fetus during antenatal ultrasound scanning. The sonologic picture may help in differentiating the lesion from many other differential diagnoses. As this usually does not alter the obstetrical management, a definite diagnosis is usually made in the postnatal period [12].

The different surgical approaches used are marsupialization, sclerotherapy, drainage, enucleation, percutaneous aspiration, and excision of the cyst with or without resection of the involved gut [13-16]. Due to high recurrence rates associated with marsupialization and drainage, complete excision of the cyst should be attempted whenever possible [14]. In adults, the cyst can often be enucleated or 'shelled out' from between the leaves of the mesentery; in children, however, bowel resection is frequently required [14,17,18]. All of our patients underwent exploratory laparotomy and complete excision of the cystic lesion with resection of the involved gut. Multiloculated cysts filled with milky fluid were found in all of our patients. The cysts were of varying sizes from the smallest approximately 8 mm in diameter to the largest approximately 9 cm in diameter. The medical literature mentions instances where laparoscopic removal of mesenteric cysts has been tried successfully, but this might have been difficult in some of our patients, especially those with large-sized chylolymphatic mesenteric cysts [19].

**Figure 3:**
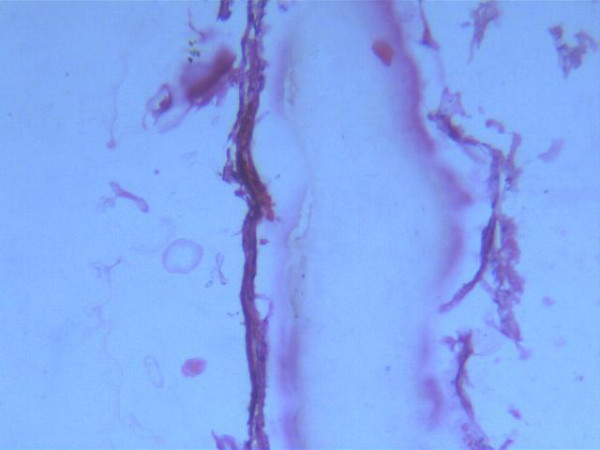
**Pediatric chylolymphatic mesenteric cyst**. Resected specimen of multiloculated chylolymphatic mesenteric cyst mimicking lymphoma.

Intra-operatively, similar findings can be seen in cystic lymphangioma, retroperitoneal cystic teratoma, caseating tubercular lymph nodes, and hydatid cysts. Even lymphoma (Figure 3) and duplication cysts may also give similar appearances. Excision biopsy is then recommended to differentiate these cases. Histopathology is confirmatory and differentiates chylolymphatic cysts from all these lesions. Cystic lymphangioma has a striking resemblance to chylolymphatic mesenteric cysts both grossly and microscopically. Some authors consider chylolymphatic mesenteric cysts to be a type of cystic lymphangioma, but the medical literature also shows some authors describing chylolymphatic cysts as a variant of mesenteric cysts [3,9,17,20]. The absence of smooth muscle and lymphatic spaces in the wall of the cyst differentiates mesenteric cysts from cystic lymphangioma [3]. During the follow-up period from 4 months to 8 years, we did not observe any recurrences, thus complete excision of the chylolymphatic cyst is curative.

## Conclusion

Although very rare, chylolymphatic mesenteric cysts should be kept in mind as one of the differential diagnoses of cystic masses of the abdomen. There are now well-established histopathological features that differentiate chylolymphatic mesenteric cysts from other cystic lesions including cystic lymphangioma. Resection of the involved gut is frequently required in children and one should not unduly prolong the surgery to avoid gut resection. Complete excision of the cyst ensures excellent prognosis and is curative.

## Consent

Written informed consent was obtained from the parents of the patients for publication of this case series and any accompanying images. A copy of the written consent is available for review by the Editor-in-Chief of this journal.

## Competing interests

The authors declare that they have no competing interests.

## Authors' contributions

MP analyzed and interpreted the patient data retrospectively. SK performed the histological examination of the kidneys, and KN and VN were major contributors in writing the manuscript. All authors read and approved the final manuscript.
